# Spatiotemporal variations and potential influencing factors of hemorrhagic fever with renal syndrome: A case study in Weihe Basin, China

**DOI:** 10.1371/journal.pntd.0011245

**Published:** 2023-04-24

**Authors:** Lingli Zhu, Liang Lu, Shujuan Li, Hongyan Ren

**Affiliations:** 1 National Institute for Nutrition and Health, Chinese Center for Disease Control and Prevention, Beijing, China; 2 State Key Laboratory of Resources and Environmental Information System, Institute of Geographic Sciences and Natural Resources Research, Chinese Academy of Sciences, Beijing, China; 3 State Key Laboratory of Infectious Disease Prevention and Control, National Institute for Communicable Disease Control and Prevention, Chinese Center for Disease Control and Prevention, Beijing, China; Beijing Children’s Hospital Capital Medical University, CHINA

## Abstract

**Background:**

Hemorrhagic fever with renal syndrome (HFRS) is a widespread zoonotic disease seriously threatening Chinese residents’ health. HFRS of Weihe Basin remains highly prevalent in recent years and attracts wide attention. With the acceleration of urbanization and related environmental changes, the interaction among anthropogenic activities, environmental factors, and host animals becomes more complicated in this area, which posed increasingly complex challenges for implementing effective prevention measures. Identifying the potential influencing factors of continuous HFRS epidemics in this typical area is critical to make targeted prevention and control strategies.

**Methods:**

Spatiotemporal characteristics of HFRS epidemic were analyzed based on HFRS case point data in Weihe Basin from 2005 to 2020. MaxEnt models were constructed to explore the main influencing factors of HFRS epidemic based on HFRS data, natural environment factors and socioeconomic factors.

**Results:**

Results showed that the HFRS epidemics in Weihe Basin were temporally divided into three periods (the relatively stable period, the rapid rising period, and the fluctuating rising period) and were spatially featured by relatively concentrated in the plains alongside the Weihe River. Landscape played controlling effect in this area while land use, vegetation and population in the area interacted with each other and drove the change of HFRS epidemic. The potential high-risk area for HFRS epidemic was 419 km^2^, where the HFRS case density reached 12.48 cases/km^2^, especially in the northern plains of Xi’an City.

**Conclusion:**

We suggested that the temporal and spatial variations in the HFRS epidemics, as well as their dominant influencing factors should be adequately considered for making and/or adjusting the targeted prevention and control strategies on this disease in Weihe Basin.

## Author summary

In recent years, the incidence rate of hemorrhagic fever with renal syndrome (HFRS) has declined in most area of China, although the acceleration of urbanization caused increasingly complicated interactions among anthropogenic activities, environmental factors, and host animals during the past decades. However, there were still some typical regions with serious HFRS epidemics in this country like the Weihe Basin in Shaanxi Province, in which more efforts should be paid for identifying the potential influencing factors and their driving mechanisms. Our study employed the MaxEnt modeling to reveal the spatio-temporal variations in the influencing factors on the epidemics of this disease from 2005 to 2020 in the Weihe Basin. We found that the landscape played controlling effect in this area while land use, vegetation and population in the area interacted with each other and drove the change of HFRS epidemic. The underling influencing factors and the stimulation of HFRS epidemic would provide detailed information for targeted strategy formulation, and the also provide reference for similar studies.

## Introduction

Hemorrhagic fever with renal syndrome (HFRS) is a widespread zoonotic disease transmitted by animals carrying Hantaviruses [1–3]. HFRS is widely prevalent in East Asian, and China is the country most seriously affected by HFRS, with about 1,557,622 HFRS cases and 46,427 deaths reported from 1950 to 2007 [4–5]. Entering the 21st century, the incidence rate has declined, and the average annual HFRS incidence rate was 0.83/10^5^ from 2011 to 2020 [6]. Since the first HFRS case was found in the Qinling Mountains of Baoji City, Shaanxi Province in 1955, Weihe Basin (also known as the Guanzhong Plain) has been one of the main HFRS epidemic areas in China [7–8]. Despite strong integrated prevention and control measures including rodent control and vaccination were taken, there is still an increasing and expanding trend in this area in recent decades [9]. A total of 9837 HFRS cases were reported in Weihe Basin from 2011 to 2015, accounting for 97.0% of the total number of cases in Shaanxi Province during the same period [10–11].

Many studies have been conducted on the epidemiological characteristics and related influencing factors of HFRS epidemic in Shaanxi Province [11–13]. These studies showed that HFRS cases in Shaanxi Province presented a spatially clustered distribution from 2011 to 2015, with high incidence clusters in Guanzhong areas, and low incidence clusters in northern and southern Shaanxi [14]. It was believed that this particular spatial distribution was closely related to environmental factors such as topography, land use, and population density [10]. Meteorological factors and vegetation may also affect HFRS epidemic by influencing the rodent activities, food availability, and contact opportunities with humans [10, 15]. Entering the 21st century, great socio-economic development brought tremendous natural and artificial environment change in this area, which may also have greatly affected the HFRS epidemics.

Previous studies have analyzed the influencing factors based on administrative scale HFRS incidence data and corresponding environmental data in this area [10–11, 13]. However, few study has been done based on long-time series HFRS case point data and fine scale influencing factors. In recent years, Ecological Niche Models are widely used in infectious disease researches to analyze fine scale influencing factors and making risk prediction, which could provide detailed information for disease control and prevention [16]. In this study, the spatial-temporal distribution characteristic and the influencing factors of the HFRS epidemic were analyzed based on HFRS case point data and MaxEnt models, which could provide referable scientific opinions for formulating epidemic prevention and control strategies.

## Material and methods

### Study area

**Fig 1:**
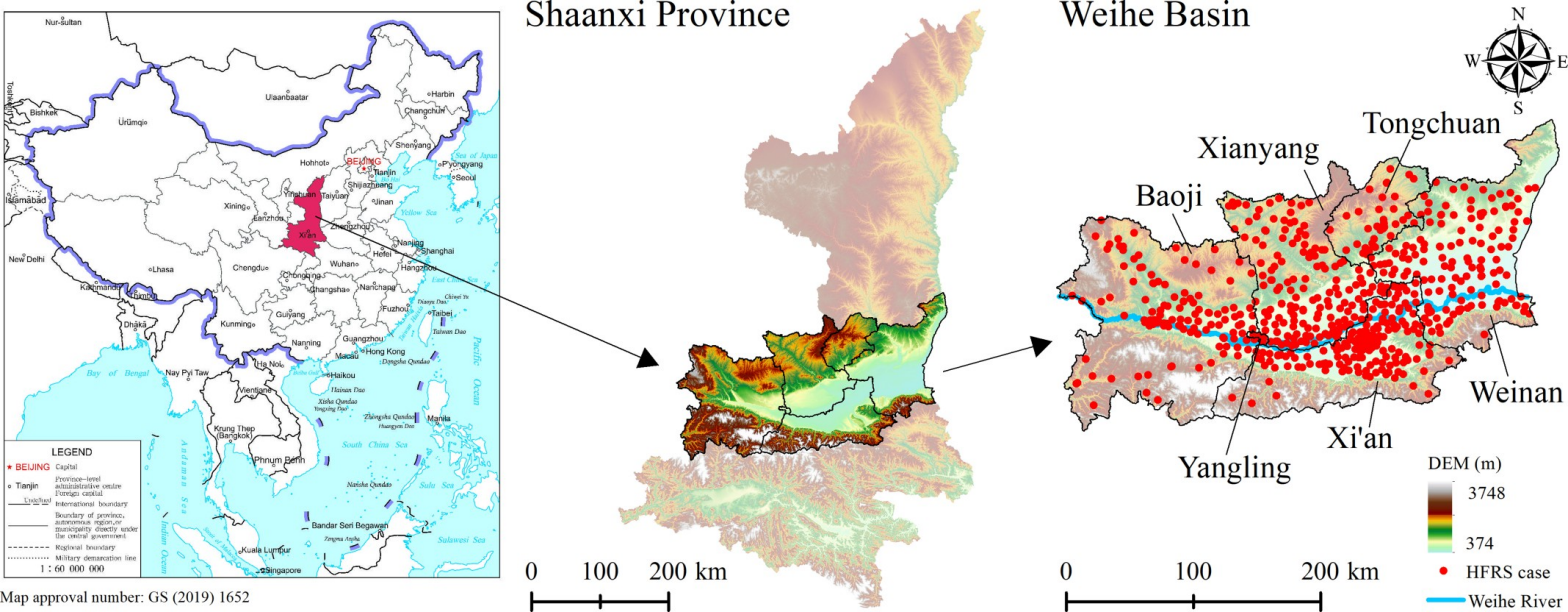
Illustration of the study area (The north of Weihe Basin is the Loess Plateau, and the south is the Qinling Mountains. The Weihe River passes through the middle of the Weihe Basin). https://www.resdc.cn/Datalist1.aspx?FieldTyepID=20,0.

Shaanxi Province is one of the provinces with the most severe HFRS epidemic in China, and the HFRS cases are mainly concentrated in Weihe Basin, including the Baoji City, Xianyang City, Xi’an City, Weinan City, Tongchuan City, and Yangling District, which was the study area of this paper (Fig 1). The continental monsoon climate in this area is distinctive: the seasons were cold, warm, dry, and wet, with precipitation decreasing from the southeast to the northwest and temperature decreasing from the center to the north and the south sides. The terrain is low in the middle and high in the north and south, with complex and diverse topography, bordering the Loess Plateau in the north and the Qinling Mountains in the south. Weihe Basin is characterized with widely distributed cultivated land, dense population, active economy, and frequent population movement. It is also the main grain and cotton production base in Shaanxi Province and the core area of population and social economy. From 2005 to 2020, a total of 26,307 HFRS cases were reported in Weihe Basin, and the cases were mainly distributed in the plains on both sides of the Weihe River at elevations below 800 m (Fig 1).

### Epidemic data and environmental data

#### Epidemic data

The data of HFRS cases in Weihe Basin from 2005 to 2020 were obtained from Chinese Center for Disease Control and Prevention. After desensitization, the information only includes gender, age, and current address. The HFRS case point data was geocoded according to the family address.

#### Environmental data

**Fig 2:**
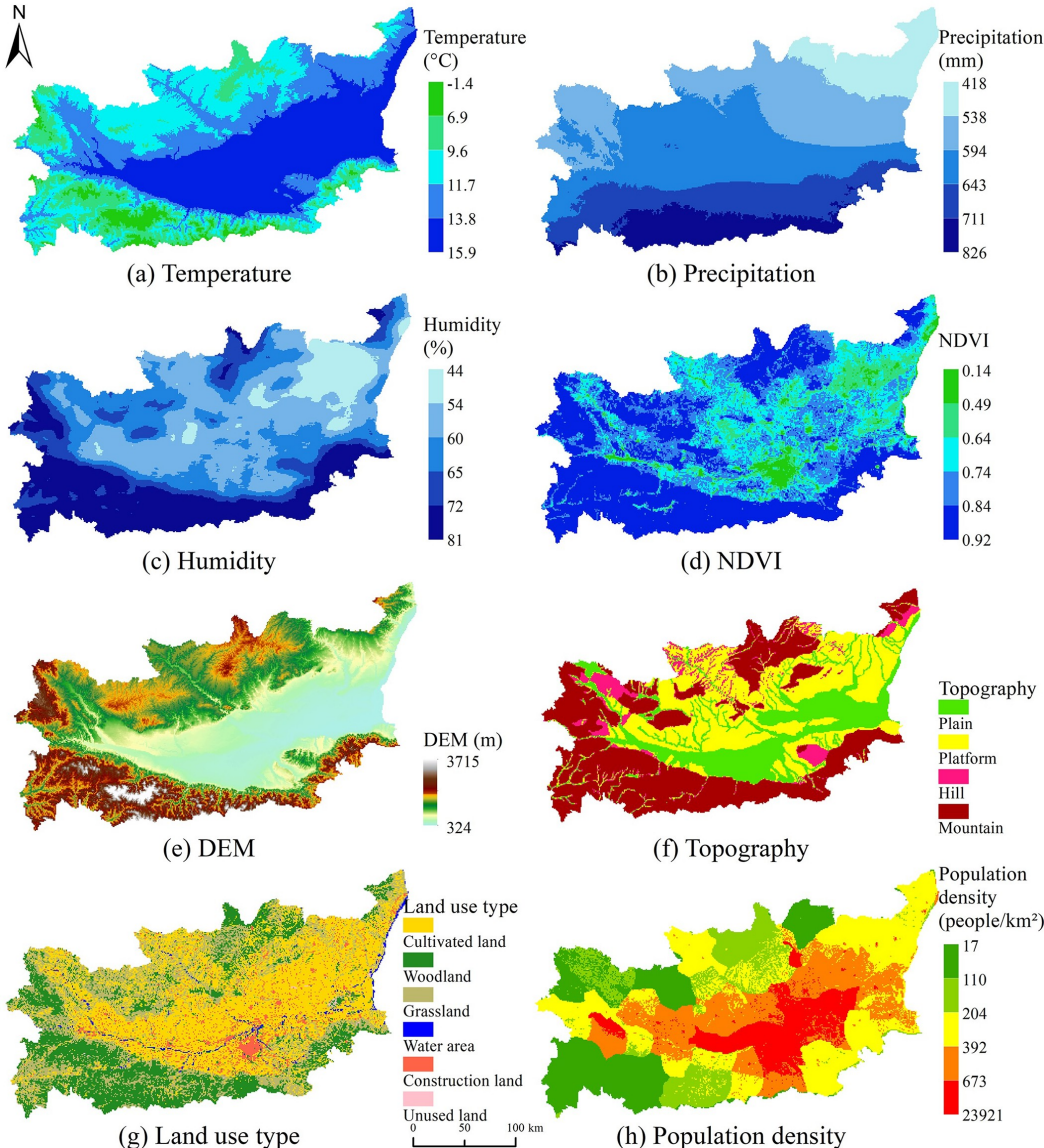
Spatial distributions of environmental and socio-economic factors in Weihe Basin of Shaanxi Province. https://www.resdc.cn/Datalist1.aspx?FieldTyepID=5,2.

According to previous studies, meteorological factors including temperature, precipitation, and humidity were closely related to host animal population growth and virus transmission [17–19]; complex landscape types have also been reported as influencing host animal habitats [13]; vegetation cover and land use were closely related to host animal survival and reproduction [20–21]; economic factors such as population density were also closely related to HFRS epidemic transmission and prevalence. According to data availability, the selection of environmental variables including temperature, precipitation, humidity, NDVI, elevation (Digital Elevation Model, DEM), topography, land use type, population density, etc., could effectively represent the climate characteristics, surface natural environmental, and socio-economic characteristics in the study area (Table 1, Fig 2). All variables covered the entire research area with a research window of 1 square kilometer, and the data were from the Resource and Environmental Data Cloud Platform of the Resource and Environmental Science Data Center of the Chinese Academy of Sciences (http://www.resdc.cn/).

**Table 1:** Variables used to construct the MaxEnt model in this study.

Factors	Data	Variable	Time Scale	Data Type
Meteorological factors	Temperature	annual average temperature	2005–2020	Continuous
	Precipitation	annual precipitation	2005–2020	Continuous
	Humidity	annual average relative humidity	2005–2020	Continuous
Landscape factors	NDVI	annual NDVI	2005–2020	Continuous
	Elevation	DEM	Permanent	Continuous
	Topography	plain, platform, hill, mountain	Permanent	Categorical
Socio-economic factors	Land use type	cultivated land, woodland, grassland, water area, construction land, unused land	2005, 2010, 2015, 2020	Categorical
	Population density	1 km grid population density data	2005, 2010, 2015, 2019	Continuous

### Research methods

#### MaxEnt model

The MaxEnt model is a general-purpose machine learning technology, based on the principle of maximum entropy, using known case points and a set of predictor variables to estimate the distribution of diseases [22–23]. In this study, it is assumed that the study area *X* is composed of a finite number of grid cells, *π* is the distribution of HFRS cases in the study area, and *π*(*x*) is the value assigned to each unit *x* by the distribution of *π*, and the sum is 1. Given the various predictor variables *f*_*j*_(*j* = 1,2,⋯,*n*) (Eqs 1 and 2), according to the approximate expected distribution of *π*, the entropy of $\tilde{\pi }$ (Eq 3) selects the distribution with the largest entropy as the HFRS optimal distribution:

$$\tilde{\pi }[f_{j}]=\frac{1}{m}\sum_{i=1}^{m}f_{j}(x_{i})$$ (1)

$$\hat{\pi }[f_{j}]=\tilde{\pi }[f_{j}]$$ (2)

$$H(\hat{\pi })=-\sum_{x\in X}\hat{\pi }(x)\hat{\ln\;\pi }(x)$$ (3)

Among them, $H(\hat{\pi })$ is the entropy of the expected distribution $\hat{\pi }$, *x*_*i*_ is the i-th unit of *X* in the study area, *m* is the number of units, *f*_*j*_ is the environment variable, and $\tilde{\pi }[f_{j}]$ is the sample point where the environment variable is at $\tilde{\pi }$ the prior mean value under the distribution.

It can be seen from the above that the probability distribution of the maximum entropy method is the same as the Gibbs probability distribution (Eq 4). Both are to maximize the similarity of all sample points and reduce the loss function (Eq 3). Because the variables used by Maxent are empirical, rather than determined actual values, over-fitting to the training data is possible. To reduce overfitting, it is necessary to appropriately relax the limits of environmental variables (Eq 6). At the same time, to enable Maxent to effectively select important environmental variables, the Gibbs distribution that minimizes the logarithmic loss and limits the excessive weight of environmental variables *λ*_*j*_ (Eq 7) is used to represent the probability distribution of the maximum entropy value:

$$q_{\lambda }(x)=\frac{e^{\lambda \cdot f(x)}}{Z_{\lambda }}$$ (4)

$$\tilde{\pi }[-\ln(q_{\lambda })]$$ (5)

Among them, *λ* is a vector representing the weight of all environmental variables, *f* is a vector of all environmental variables, and *Z*_*λ*_ is a standardized constant to ensure that the sum of *q*_*λ*_ is 1:

$$|\hat{\pi }[f_{j}]-\tilde{\pi }[f_{j}]|\leq \beta_{j}$$ (6)

$$\tilde{\pi }[-\ln(q_{\lambda })]+\sum_{j}\beta_{j}|\lambda_{j}|$$ (7)

Among them, *β*_*j*_ is a constant, $\tilde{\pi }[-\ln(q_{\lambda })]$ is a loss function, and ∑_*j*_*β*_*j*_|*λ*_*j*_| represents the restriction on the weight of environmental variables.

#### Model construction

**Fig 3:**
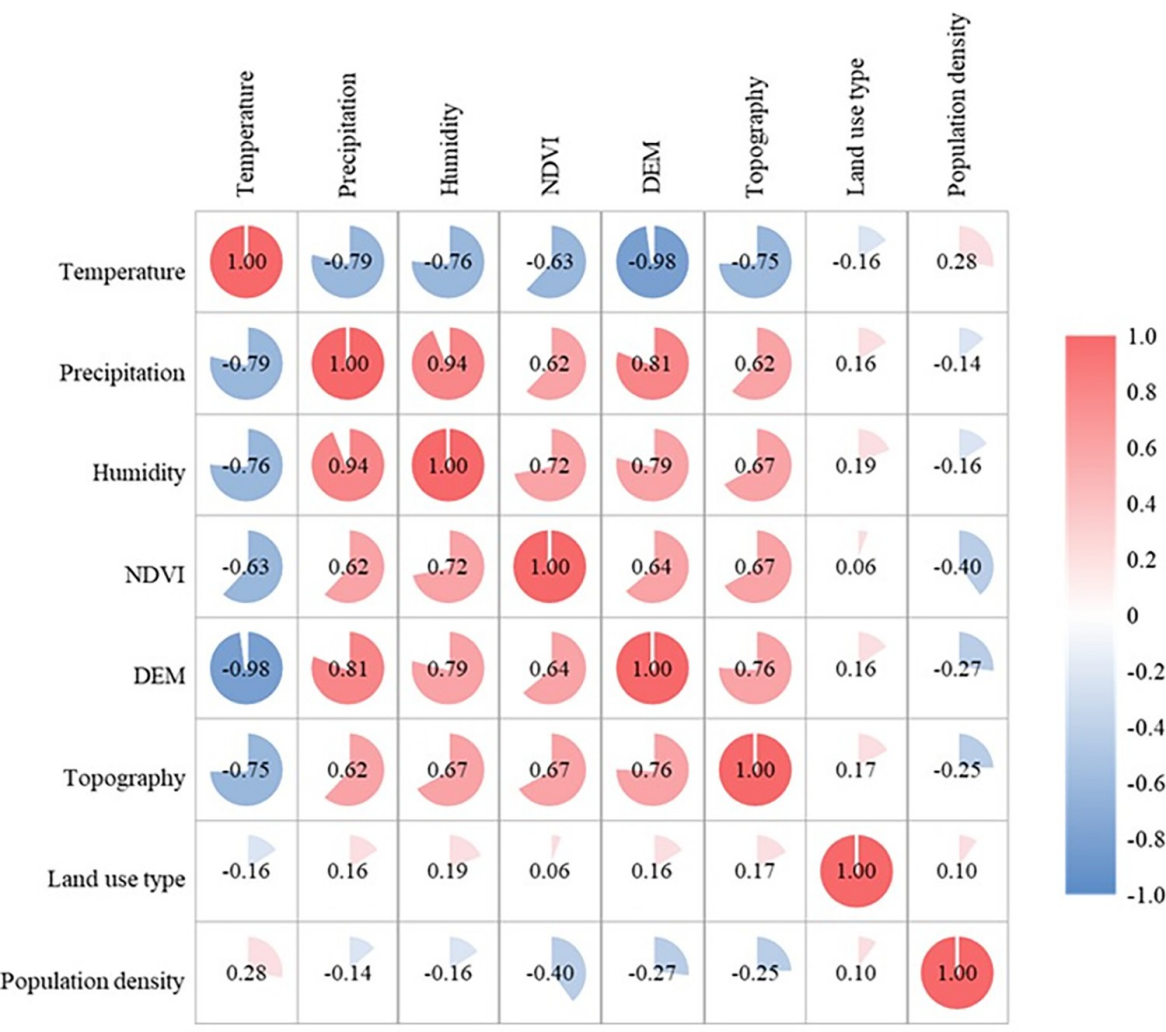
Correlation coefficient between environmental variables.

If there is a high correlation between variables, it may affect the output results of the MaxEnt model [24–25]. To a certain extent, the environmental variables involved in the construction of MaxEnt model have a collinearity problem. This study first calculated the correlation coefficient between all environmental variables (Fig 3). Since the correlations between temperature and DEM, precipitation and humidity were significant (P<0.01), the best model was selected according to the model fitting results. The finally selected environmental variables, including precipitation, NDVI, DEM, topography, land use, and population density.

#### Model evaluation

To evaluate the results of the model, this study divided the HFRS case distribution data into two parts. We used 75% of the HFRS case data as the training sample, and the remaining 25% of the HFRS case data were used as the test sample. At the same time, in the modeling process, the training samples and test samples in the modeling samples were combined with 10,000 background points to draw the receiver operating characteristic curve (ROC), and the value of area under the curve (AUC) was calculated. Each value of the predicted result was used as a possible judgment threshold by the ROC curve, and the corresponding sensitivity and specificity were calculated. The false positive rate (specificity) was plotted as the abscissa, and the true positive rate (sensitivity) was plotted as the ordinate. The AUC value could be used as a measure of the prediction accuracy of the model, and its value ranged from 0 to 1. The larger the value, the stronger the model’s judgment. It is generally considered that the diagnostic value is low when AUC is between 0.5 and 0.7, medium when AUC is between 0.7 and 0.9, and high when AUC is greater than 0.9.

## Results

### Temporal characteristics of HFRS epidemic

**Fig 4:**
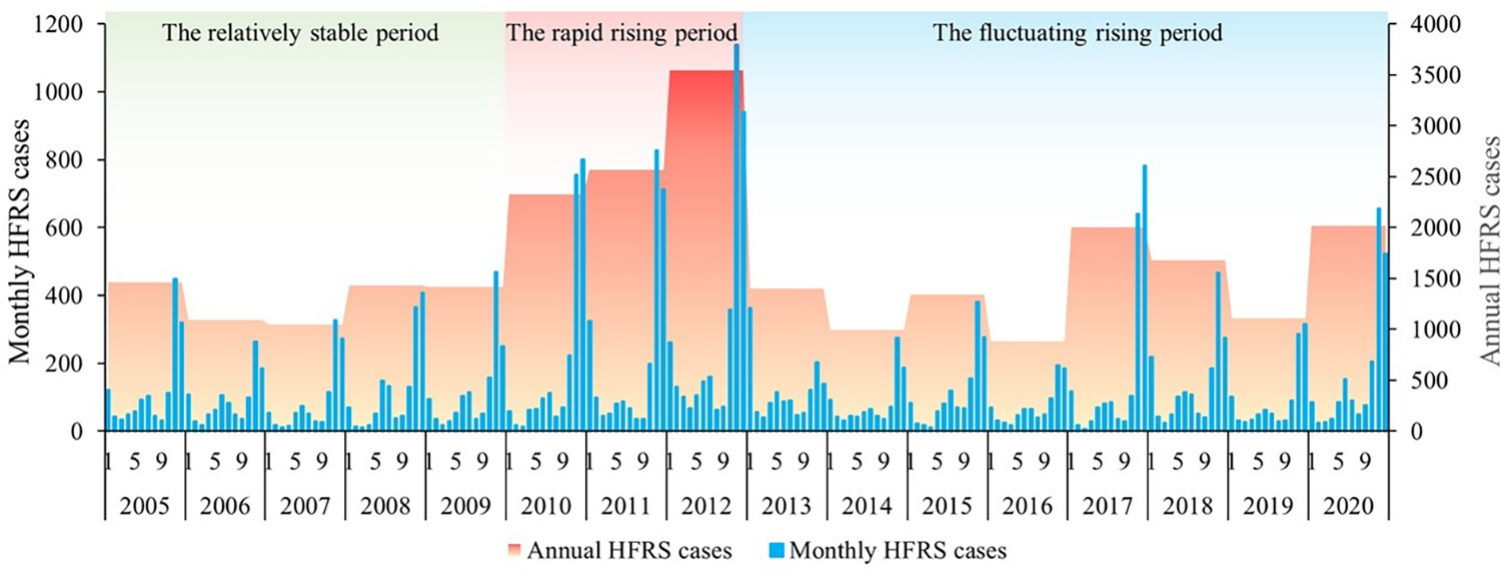
The number of annual and monthly HFRS cases in Weihe Basin of Shaanxi Province from 2005 to 2020.

From 2005 to 2020, the cumulative number of reported HFRS cases in Weihe Basin was 26,307. From 2005 to 2009, the average annual HFRS cases remained at about 1,289 (standard deviation: 202.03; range: 1047 to 1460; 95% CI: 1038.55, 1540.25), and the epidemic situation remained relatively stable. From 2010 to 2012, the annual HFRS cases increased sharply to 3,542 (mean:2811; standard deviation: 644.81; range: 2323 to 3542; 95% CI: 1209.21, 4412.79). From 2013 to 2020, the HFRS epidemic fluctuated and increased, and the annual HFRS cases fluctuated in the range of 885 to 2,014 (mean:1428; standard deviation: 435.07; 95% CI: 1064.65, 1792.10). In addition, the HFRS epidemic in Weihe Basin presented an obvious “double-peak” characteristic (Fig 4), with a large peak in autumn and winter concentrated from October to January and a small peak in summer concentrated in June to July. The large peak accounted for 71.9% (non-large peak was 28.1%) of the total number of HFRS cases in the year. The results showed that the HFRS epidemic in Weihe Basin from 2005 to 2020 was split into the relatively stable period, the rapid rising period, and the fluctuating rising period, with significant seasonal characteristics of a large peak in autumn and winter and a small peak in summer.

### Spatial-temporal characteristics of HFRS epidemic

**Fig 5:**
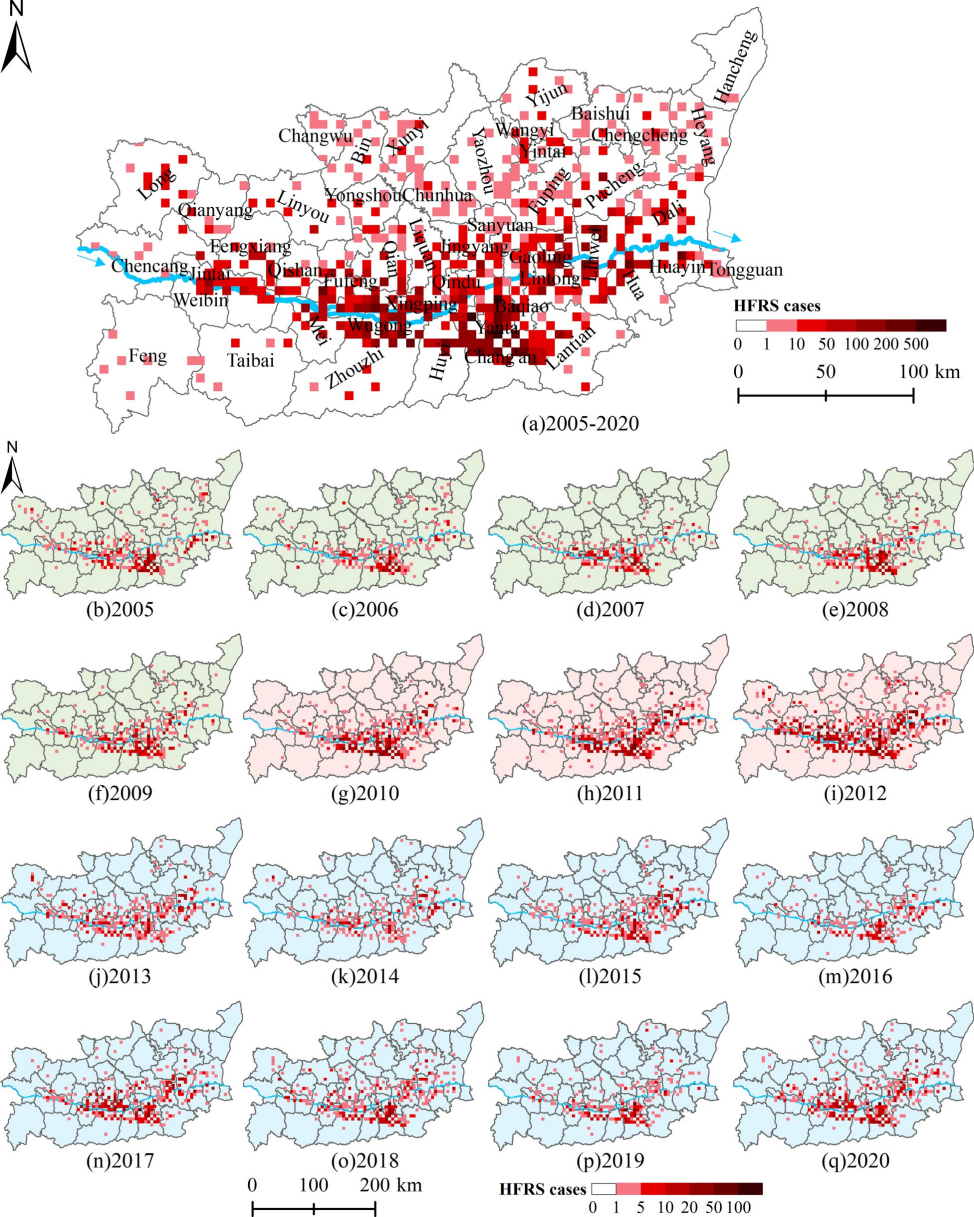
The number of HFRS cases at the 5-kilometer grid scale in Weihe Basin from 2005 to 2020. https://www.resdc.cn/Datalist1.aspx?FieldTyepID=20,0.

The HFRS epidemic showed different spatial distribution characteristics in different epidemic periods (Fig 5). The grids with high HFRS cases mainly distributed along with Weihe River. From 2005 to 2009, the HFRS epidemic mainly occurred in Zhouzhi County, Huyi District, and Chang’an District of Xi’an City. Since 2010, the HFRS epidemic has gradually spread downstream of the Weihe River. In 2012, the overall epidemic situation was very serious, especially in Chang’an District of Xi’an City, Linwei District of Weinan City, and Fufeng County of Baoji City with more than 300 cases. After 2013, the overall trend eased, but the downstream situation was not optimistic, especially in Lintong District of Xi’an City and Linwei District of Weinan City. The results showed that the HFRS epidemic in Weihe Basin was mainly distributed in the plain areas, and had a tendency to spread to the north bank and downstream of the Weihe River.

### Influencing factors of the HFRS epidemic in different epidemic periods

According to the three-epidemic period of HFRS in Weihe Basin: the relatively stable period (2005–2009), the rapid rising period (2010–2012) and the fluctuating rising period (2013–2020), MaxEnt models for four time periods: 2005–2009, 2010–2012, 2013–2020 and 2005–2020 were constructed to compare the influencing factors respectively. The fitting results were shown in Table 2. The AUC values of the MaxEnt models for the three epidemic periods were 0.80, 0.81, and 0.80, respectively, which were slightly higher than the entire epidemic period with an AUC value of 0.79. The results indicated that the MaxEnt models of different epidemic periods fitted better and performed better in explaining the changes of influencing factors of HFRS epidemic.

**Table 2:** The fitting results of four MaxEnt models.

	The relatively stable period (2005–2009)	The rapid rising period (2010–2012)	The fluctuating rising period (2013–2020)	The entire period (2005–2020)
Training AUC	0.81	0.82	0.81	0.80
Test AUC	0.80	0.81	0.80	0.79

In terms of the contribution rate of each environmental variable to the MaxEnt model (Table 3), topography was the key controlling factor of HFRS epidemic in this area, with contribution rate more than 30%. Land use type was also the main factor imposing continuous and steady effect on HFRS epidemic. The contribution rate of NDVI rose sharply from 9.41% (the relatively stable period) to 14.36% (the rapid rising period) and 34.07% (the fluctuating rising period), while the contribution rate of population density dropped rapidly from 23.65% (relatively stable period) and 19.86% (rapid rising period) to 0.97% (fluctuating rising period). The results showed that the HFRS epidemic in Weihe Basin from 2005 to 2020 was mainly influenced by topography and NDVI, as well as land use type and population density.

**Table 3:** Contribution rate (%) of environmental variables to MaxEnt model.

Variable	The relatively stable period (2005–2009)	The rapid rising period (2010–2012)	The fluctuating rising period (2013–2020)	The entire period (2005–2020)
Precipitation	3.15	5.41	6.73	5.09
NDVI	9.41	14.36	34.07	27.52
DEM	1.56	1.75	1.07	1.40
Topography	35.78	30.04	30.99	34.76
Land use type	26.45	28.58	26.17	22.72
Population density	23.65	19.86	0.97	8.51

**Fig 6:**
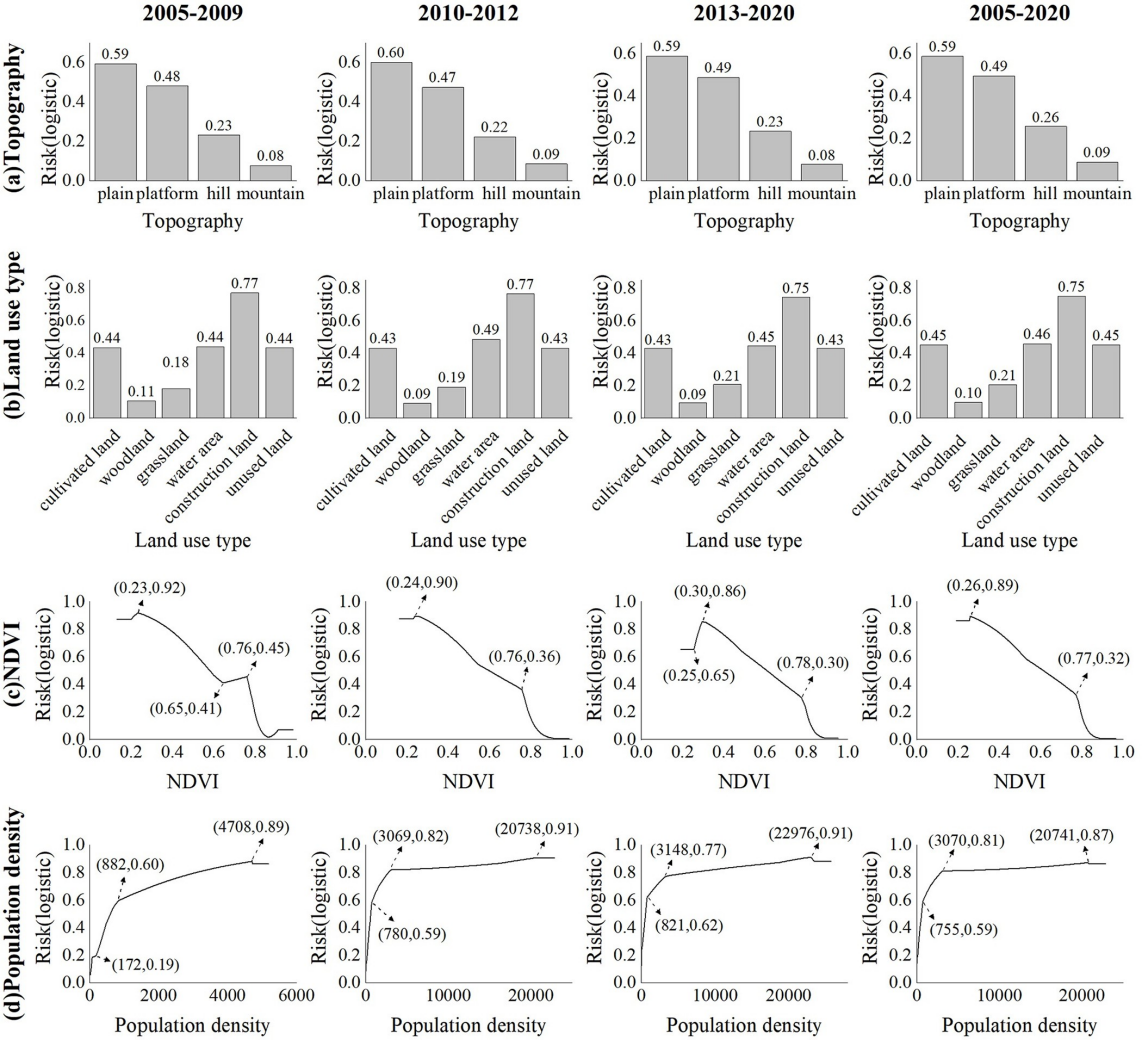
The response curves of the major environmental factors to the risk of HFRS epidemic. **a** and **b** were the risk values of different topography and land use on the spread of HFRS epidemic, respectively. **c** and **d** were the response curve of NDVI and population density to the spread of HFRS epidemic, respectively.

Fig 6 plotted the response curves of the major environmental factors (the four environmental factors with highest contribution rate) to the risk of HFRS epidemic in the four time periods. According to the risk values of different landform types on the spread of HFRS epidemic (Fig 6A), the plain was the main risk area, with risk values above 0.59 in all four epidemic periods; the followed was the platforms, with risk values all above 0.47; hills and mountains had the lowest risk of epidemic transmission. In terms of land use types (Fig 6B), construction land was the main risk land use type for HFRS epidemic transmission, with risk values above 0.75 in all four epidemic periods; cultivated land, water area, and unused land had similar transmission risks, with risk values around 0.44; grassland and woodland had the lowest transmission risks. Fig 6C showed the impact of different vegetation index values on the risk of HFRS epidemic transmission. During the fluctuating rising period (2013–2020), there was lower risk in the low-value area (NDVI<0.25), while there was higher risk in the low-value area (NDVI<0.25) during the other period. Except that, the trend were similar, which showed a decreasing trend after reaching the peak risk. Fig 6D showed the impact of different population densities on the risk of epidemic transmission. The trends of the response curves in the four time periods were mainly same, which showed an upward trend and then remained stable. The main difference was that the inflection point in 2005–2009 was 172 people/km^2^, and in 2010–2012 and 2013–2020 they were 780 people/km^2^ and 821 people/km^2^, respectively. When population density increased more than 3000 people/km^2^ and below around 20000 people/km^2^, the risk kept at a high level and increased at a low rate. It can be seen that the risk values of topography, land use and NDVI for the spread of HFRS epidemics in different epidemic periods had small changes, while population density varied greatly in the risk value ranges of different epidemic periods.

### HFRS epidemic risk simulation

**Fig 7:**
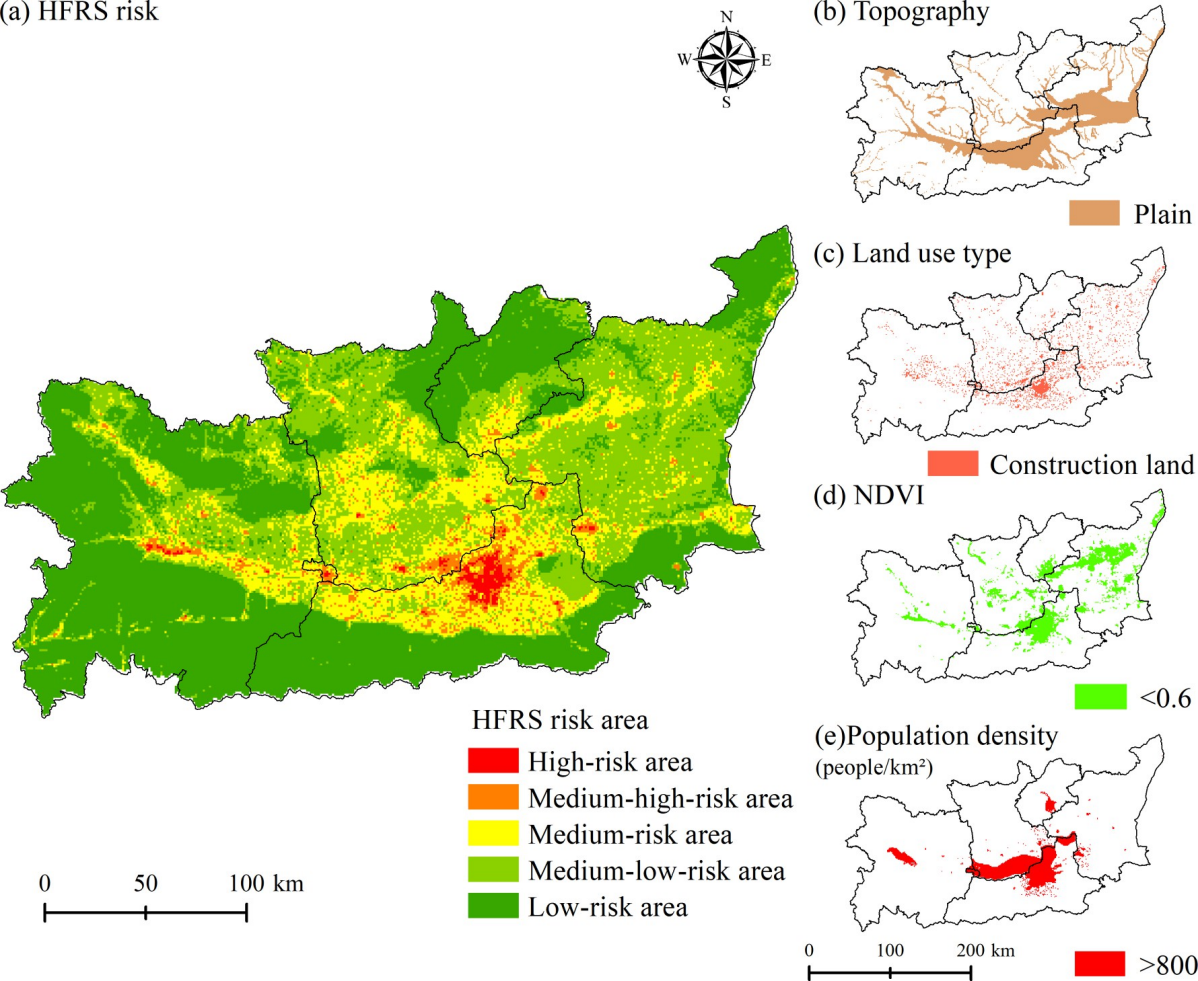
Potential risk area of HFRS epidemic and spatial distribution of major environmental factors in 2015. https://www.resdc.cn/Datalist1.aspx?FieldTyepID=20,0.

The potential HFRS case distribution probability was stimulated and expressed as the risk rate, which was between 0 and 1. With a higher risk rate, the area is with a higher possibility to have a HFRS case. To make it easier to define the risk level, the risk area of HFRS epidemic in Weihe Basin was divided into the high-risk area (risk rate between 0.8–1), medium-high-risk area (risk rate between 0.6–0.8), medium-risk area (risk rate between 0.4–0.6), medium-low-risk area (risk rate between 0.2–0.4) and low-risk area (risk rate between 0–0.2) (Fig 7). The high-risk area was mainly concentrated in the plain area of Xi’an City, including Xincheng District, Beilin District, Lianhu District, Yanta District, Weiyang District, Baqiao District, and other districts and counties. The medium-high-risk area was mainly distributed around the high-risk area, including the northern part of Chang’an District, Huyi District, Zhouzhi County, and Lantian County, the southern part of Qindu District, Weicheng District, Xingping City, Gaoling District, Lintong District, and Yangling District, and the junction of Chencang District, Weibin District, and Jintai District.

The correspondence between the number of HFRS cases and the level of risk zoning in Weihe Basin showed that the proportion of HFRS cases in the high-risk area and the medium-high-risk area accounted for 47.4% of the total number of HFRS cases, while the risk area accounted for only 4.39% of the total area (Table 4). The case density in the high-risk area reached 12.48 cases/km^2^ and there were about 3.2 million persons in this area, at the same time, in the medium-high-risk area the case density reached 3.62 cases/km^2^ and there were about 2.68 million persons in the area.

**Table 4:** The correspondence between the number of HFRS cases and the level of risk zoning in Weihe Basin.

Risk area	Number of HFRS cases	Proportion of total cases (%)	Area (km^2^)	Risk area ratio (%)	HFRS density (HFRS cases/km^2^)	Population (10,000 persons)
High-risk area	5231	19.88	419	0.77	12.48	320
Medium-high-risk area	7238	27.52	1974	3.62	3.67	268
Medium-risk area	8540	32.46	9447	17.34	0.90	513
Medium-low-risk area	4904	18.64	17783	32.64	0.28	708
Low-risk area	394	1.5	24860	45.63	0.02	473

**Fig 8:**
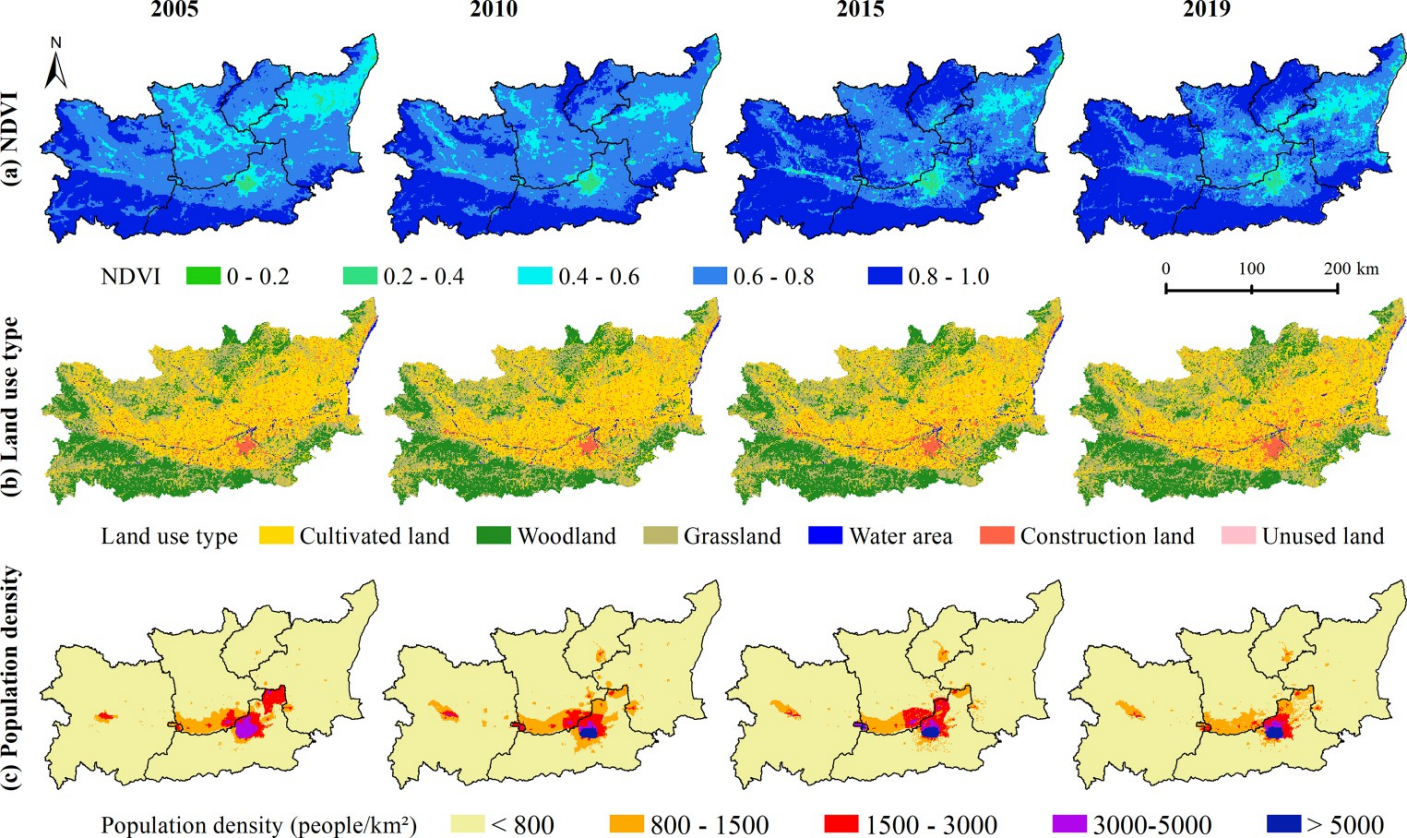
Spatial distribution map of NDVI, land use type and Population density in 2005, 2010, 2015 and 2019. https://www.resdc.cn/Datalist1.aspx?FieldTyepID=20,0.

Combining the spatial distribution maps of NDVI, land use and population density in 2005, 2010, 2015, and 2019 (Fig 8), the high-risk area and medium-high-risk area were mainly distributed in areas with low vegetation cover and dense population distribution, and land use was mostly construction land and cultivated land. The high-risk area and medium-high-risk area were superimposed areas of the risk thresholds of the main influencing factors of topography, land use, NDVI, and population density, and this characteristic became more obvious over time. The results showed that although the high-risk area and the medium-high-risk area accounted for a relatively small area, the potential HFRS epidemic risk was very high, especially in the northern plains of Xi’an City, where NDVI was below 0.6 and population density was higher than 800 people/km^2^.

## Discussion

As a zoonotic disease, HFRS epidemic is greatly affected by natural environmental and socio-economic factors and presents typical regional characteristics [26–29]. Although many efforts have been made to reduce HFRS infection in China, there are still areas with high epidemic incidence. Weihe Basin is a typical HFRS epidemic area with fluctuating high incidence. Revealing spatial-temporal distribution change and its influencing factors could provide effective information for disease prevention and control. In this study, we analyzed the spatiotemporal distribution characteristics of the HFRS epidemic in Weihe Basin from 2005 to 2020, and built MaxEnt models to identify the influencing factors and simulate the potential risk areas.

HFRS epidemic of Weihe Basin from 2005 to 2020 was split into the relatively stable period (2005–2009), the rapid rising period (2010–2012), and the fluctuating rising period (2013–2020). The number of HFRS cases reached the highest in 2012 with 3,542 cases. The current HFRS epidemic in Weihe Basin was still on an upward path, which may be related to the 5-10-year epidemic cycle of HFRS [30–31]. At the same time, HFRS cases occurred in every month of each year, with a large peak in autumn and winter and a small peak in summer. The seasonal characteristic might be related to the human activities and the dominant rodent species in the study area: *Apodemus agrarius*, *Rattus norvegicus*, and *Mus musculus* [32] in the area. As an important commercial grain producing areas, the grain output of Weihe Basin accounts for more than 50% of the whole province [33]. The crop planting pattern in Weihe Basin is winter wheat and summer maize planted alternately throughout the year, and the harvest seasons are in June and October respectively. In the harvest season, sufficient food increases rodent density and its activities; at the same time, farmers taking farm work get more opportunities to contact with rodents when the crops are mature and harvested, which increased HFRS infection [27]. Since Weihe Basin is still in a high-incidence epidemic year, we suggested that local authorities should pay more attention to the prevention and control of the HFRS epidemic, and strengthen the monitoring of rodent conditions and rodent control in the wild before the peak of HFRS epidemic.

The spatial-temporal distribution of HFRS cases presented a concentrated trend in the plain area, which was consistent with the previous study [11]. More than 90% of HFRS cases were distributed in the plain areas on both sides of the Weihe River with an altitude less than 800 m, which indicated the control effects of topography (contribution rate in the whole period: 34.76%). Unlike the topography as invariable factor, land use, vegetation and population density are the prominent variable factors affecting HFRS epidemic, which interact with each other and jointly constitute the occurrence environment for HFRS cases. Compared with vegetation and population density, land use posed a steady and sustained impact on HFRS, with contribution rate about 26% in different periods. Construction land had the highest HFRS transmission risk, which was consistent with the other study [11]. From 2005 to 2020, the scope of construction land in the plain area expanded, with surrounding cultivated land converted into construction land (Fig 8B). Those converted areas were usually distributed on the rural-urban fringes, where congregated with densely floating population and human-rodent contact opportunities [34]. Cultivated land was also with high epidemic risk, since it provided sufficient food and habitat for rodents and working place for farmers engaged in agricultural production [35]. Therefore, we recommend that vaccination and knowledge dissemination on prevention and control need to be done for key groups such as farmers, migrant workers, and construction workers.

NDVI and population density were important influencing factors with great variation in different epidemic periods. The contribution rate of NDVI increased sharply from 9.41% (the relatively stable period) and 14.36% (the rapid rising period) to 34.07% (the fluctuating rising period), indicating that vegetation cover played an important role in the spread of HFRS epidemic. Combining with the response curve of NDVI to the spread of the HFRS epidemic, it could be seen that the risk of HFRS transmission was higher in the low NDVI area than that in the high NDVI area. Corresponding with the land use types, the areas with low NDVI values (NDVI<0.6) were mainly construction land and cultivated land (Fig 8).

Population density had a positive effect on the spread of HFRS epidemic in all three epidemic periods, with contribution rate reaching 23.65% and 19.86% in the relatively stable period and the rapid rising period, and dropping into 0.97% in the fluctuating rising period. Contrary to the biphasic effect between HFRS incidence and population density in the other studies [36–37], population density kept a positive effect in this study. As population density increases, the HFRS transmission risk increases accordingly. The HFRS epidemic risk increased rapidly when the population density was among 800 people/km^2^ to 3000 people/km^2^, and this specific population density was spatially distributed in the urban-rural ecotone (Fig 8). The speed of urbanization in the surrounding rural areas has been accelerated, with the newly built industrial parks expanding on a large scale, which resulted in population increase and also the fragmentation of the habitat of rodents. Seriously, the living environment of rodents has been destroyed, and its range of activities has spread to the human living environment, resulting in the spread of the virus. Also, we analyzed the possible reasons for the rapid decline in the contribution of population density during the fluctuating rising period. By comparing the population density data for 2005, 2010 and 2015 (Fig 7), the areas with obvious changes in population density were mainly distributed in areas with more than 800 people/km^2^. These areas correspond to the urban interior. Combined with the response curve (Fig 5), it can be found that when the population density increased to a certain threshold, the impact on the spread of HFRS epidemic tended to be flat. At this time, the increase of population density in cities and towns would not increase the risk of epidemic spread. At the same time, we also noticed that the contribution rate of NDVI had greatly improved during this period, which also reflected that vegetation played a greater role in this period from the side. The low value area of NDVI had higher transmission risk, and its corresponding land use types were mainly cultivated land and construction land, which were mainly distributed at the junction of urban and rural areas, with relatively high transmission risk.

In addition, the potential high-risk areas for the HFRS epidemic in Weihe Basin were mainly distributed in the northern plains of Xi’an City with low vegetation cover and dense population distribution. Xi’an City, as the city with the highest level of economic development in Weihe Basin, had more than 50% of the total number of HFRS cases in the study area. The city center of Xi’an City, including Xincheng District, Beilin District, and Lianhu District, were also a potential high-risk area for HFRS epidemic, which may be related to the dense population and frequent human activities such as large-scale project construction, coupled with the integration of Xi’an and Xi’an metropolitan area. The increase of vulnerable populations such as construction workers and floating populations has led to a consistently high level of local outbreaks. Therefore, we suggested that while ensuring the orderly and efficient development of the local economy, the prevention and control of the HFRS epidemic needs to be done effectively.

The following limitations of this study should be mentioned. Firstly, we used the family address of HFRS case rather than the infection location to locate the case point, which would have a certain impact on the analysis results of influencing factors. Secondly, this study did not include data of rodent density and virus-carrying rate due to data availability, which may provide opportunity to explain the mechanism of interaction among rodents, environmental factors and HFRS infection. Thirdly, as a traditional HFRS epidemic area, vaccination had been implemented in Shaanxi Province since 2004 [38]. However, due to the accessibility, the vaccination data was not used in analyzing the influencing factors and simulating potential risks, which might affect the results. In addition, we simulated the extent of potential risk areas of HFRS epidemic, which could provide useful information for developing targeted measures for HFRS epidemic outbreak prevention and control. In the future, we will use future scenario data to predict the HFRS epidemic, and provide accurate information for the prevention and control of HFRS epidemics.

## Conclusions

The HFRS epidemic in Weihe Basin from 2005 to 2020 was split into three epidemic periods, and HFRS cases were mainly distributed in the plains on both sides of the Weihe River. The spatiotemporal distribution characteristic of HFRS in this area was determined by topography, land use type, vegetation and population density, while vegetation and population density played different roles in different epidemic periods. We suggested that more attention should be paid to the densely populated plain areas on both sides of the Weihe River, especially the areas where cultivated land and construction land were widely distributed. Key population groups such as farmers and workers should be vaccinated and popularized with the knowledge of prevention and treatment. This study provided valuable clues for local health departments to design and implement effective prevention and control measures to control the HFRS epidemic.

## Ethics statement

These data collection is part of routine public health surveillance and is exempt from institutional review board evaluation. Ethical approval for this study was not required in accordance with local legislation and national guidelines.

## Supporting information

S1 Data Epidemic data and environmental data. **Folder 00HFRS cases in S1 Data**: The number of HFRS cases at the 5-kilometer grid scale in Weihe Basin from 2005 to 2020. **Folder 01Temperature in S1 Data**: Annual average temperature raster data from 2005 to 2020. **Folder 02Precipitation in S1 Data**: Annual precipitation raster data from 2005 to 2020. **Folder 03Humidity in S1 Data**: Annual average humidity raster data from 2005 to 2020. **Folder 04NDVI in S1 Data**: Annual NDVI raster data from 2005 to 2020. **Folder 05DEM in S1 Data**: Elevation raster data. **Folder 06Topography in S1 Data**: Topography raster data. **Folder 07Land use type in S1 Data**: Land use raster data in 2005, 2010, 2015, and 2020. **Folder 08Population density in S1 Data**: Population density raster data in 2005, 2010, 2015, and 2019.
(RAR)
